# Great chemistry between us: The link between plant chemical defenses and butterfly evolution

**DOI:** 10.1002/ece3.7673

**Published:** 2021-05-27

**Authors:** Corné F. H. van der Linden, Michiel F. WallisDeVries, Sabrina Simon

**Affiliations:** ^1^ Biosystematics Group Wageningen University & Research Wageningen The Netherlands; ^2^ De Vlinderstichting/Dutch Butterfly Conservation Wageningen The Netherlands; ^3^ Plant Ecology and Nature Conservation Group Wageningen University & Research Wageningen The Netherlands

**Keywords:** butterfly assemblages, chemical defense, coevolution, ecological communities, macroevolution, plant–insect interaction

## Abstract

Plants constantly cope with insect herbivory, which is thought to be the evolutionary driver for the immense diversity of plant chemical defenses. Herbivorous insects are in turn restricted in host choice by the presence of plant chemical defense barriers. In this study, we analyzed whether butterfly host–plant patterns are determined by the presence of shared plant chemical defenses rather than by shared plant evolutionary history. Using correlation and phylogenetic statistics, we assessed the impact of host–plant chemical defense traits on shaping northwestern European butterfly assemblages at a macroevolutionary scale. Shared chemical defenses between plant families showed stronger correlation with overlap in butterfly assemblages than phylogenetic relatedness, providing evidence that chemical defenses may determine the assemblage of butterflies per plant family rather than shared evolutionary history. Although global congruence between butterflies and host–plant families was detected across the studied herbivory interactions, cophylogenetic statistics showed varying levels of congruence between butterflies and host chemical defense traits. We attribute this to the existence of multiple antiherbivore traits across plant families and the diversity of insect herbivory associations per plant family. Our results highlight the importance of plant chemical defenses in community ecology through their influence on insect assemblages.

## Graphical abstract

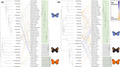

## 1 INTRODUCTION

Plants and arthropod herbivores share a 420 million‐year history of antagonistic coevolution (Labandeira, 1998). Plants are under constant pressure of herbivory and have developed numerous ways to defend themselves against, for example, herbivorous insects. Here, chemical defenses, composed of secondary metabolites, form one of the most complex defense mechanisms (Ehrlich & Raven, 1964; Wink, 2003, 2018; Wink et al., 1998). The large diversity of plant secondary metabolites is considered an evolutionary result of millions of years of herbivorous pressure, continually acting as the evolutionary driver of antiherbivore defenses (Farrell et al., 1991; Schoonhoven et al., 2005). Secondary metabolites are compounds not directly involved in growth, reproduction, and cellular maintenance of plants but play a prominent role in other processes such as antiherbivore defense (Schoonhoven et al., 2005; Wink, 2018). Major similarities and differences of secondary metabolites are generally conserved at plant family level (Becerra, 2007; Farrell et al., 1991; Volf et al., 2017; Wink, 2008). Herbivorous insects and angiosperm plants, both highly species‐rich groups, interact through an extraordinary diversity of ecological interactions. Many aspects of insect–plant interactions are poorly understood, especially the implications for ecological and evolutionary patterns of both groups. There are a few exceptions, such as for the well‐studied butterflies (Lepidoptera: Papilionoidea) (Ehrlich & Raven, 1964; Ferrer‐Paris et al., 2013; Fordyce, 2010; Maron et al., 2019). Butterflies have close associations with their larval host plants, the majority of species feeding only on a small group of plant species during their larval stage. The evolution of these ecological relationships between butterflies and their hosts is thought to be explained, at least in part, by the presence of shared plant chemical defenses (Schoonhoven et al., 2005).

Ehrlich and Raven (1964) identified secondary metabolites as a form of antiherbivore defense, but more importantly gave rise to the “escape and radiate” scenario of speciation driven by chemical defenses (Endara et al., 2017; Farrell et al., 1991; Maron et al., 2019; Thompson, 1994). In this scenario, adaptation of insect herbivores to plant chemical defenses acts as a driver for the evolution of novel plant chemical defenses. This allowed for subsequent plant radiation, with herbivores coevolving and following plant diversification (Ehrlich & Raven, 1964; Thompson, 1994). Ehrlich & Raven's coevolution scenario implies a strong phylogenetic pattern in host plant selection, with suitable hosts being closely related and butterfly lineages following plant lineages. However, in contrast to this hypothesis, the host plant range of many butterflies shows a high degree of phylogenetic dissimilarity, that is, a large portion of butterflies use multiple hosts of distantly related plant families (Bink, 1992; Tolman & Lewington, 2008; Tshikolovets, 2011). This indicates that there are additional factors that, along with phylogenetic conservatism, play a deterministic role in butterfly–host interaction patterns. Ecological theory suggests that similarity of host functional traits might better explain congruence between butterflies and their host plants than phylogenetic relationships alone. Such host plant similarity may be in the form of ecological niches, structural defenses, biotic defenses, seasonal palatability, nutritional composition, or secondary metabolite composition (Becerra, 1997; Endara et al., 2017; Murphy & Feeny, 2006; Pearse & Hipp, 2009; Rapo et al., 2019). Indeed, there is growing evidence for the importance of chemical defenses in shaping herbivore assemblages and driving coevolutionary interactions of butterflies and their host plants (Ferrer‐Paris et al., 2013; Fordyce, 2010; Janz et al., 2001; Wahlberg, 2001). Studies of small interaction networks imply that plant–herbivore interaction patterns (Endara et al., 2017, 2018) and evolutionary relationships (Becerra, 1997; Wahlberg, 2001) correspond more strongly with shared host plant chemical defenses than with the phylogenetic relationships of the host plants. Nevertheless, widespread investigation is still required for confirmation at higher phylogenetic levels and macroevolutionary scales (Agrawal, 2007).

The butterfly fauna of northwestern Europe presents a suitable study case here, because it is one of the best researched of insect groups; their interactions with plants, distribution, and taxonomic relationships are well known. We examine relationships of butterflies with their host plant families using the insect–plant interactions of the northwestern European butterflies, comprising 145 species of six different butterfly families. These families are the Hesperiidae (skippers), Riodinidae (metalmarks), Lycaenidae (blues, coppers, and hairstreaks), Nymphalidae (brush‐footed butterflies), Papilionidae (swallowtails), and Pieridae (whites and sulphurs). We evaluate the relationship of butterflies and plant chemical defenses by characterizing plant family chemical defenses through the aggregation of literature sources on secondary metabolite composition. We use matrix correlation and cophylogenetic statistics to evaluate the following: (a) if plant chemical defenses, rather than host plant phylogenetic relationships, predict butterfly–host plant use, and (b) if cophylogenetic interactions with plant chemical defenses are present at a macroevolutionary scale. We expect host plant chemical defenses to be an important predictor for butterfly–host use and expect to detect cophylogenetic interactions between butterflies and host plant chemical defenses.

## 2 METHODS

### 2.1 Compiling butterfly–host interactions

The interaction network of the butterfly species of northwestern Europe and their host plants was used as a case study. An extensive herbivore–plant interaction database was compiled from numerous literature sources on host use of the European butterfly species (Bink, 1992; Tolman & Lewington, 2008; Tshikolovets, 2011). Although referred to throughout the text as butterfly–plant interactions, it is important to note that the interactions examined here consist of herbivory interactions between immature stages of butterflies and their host plants (i.e., deposited eggs & caterpillar folivory). Host associations of 145 butterfly species, comprising six families (Hesperiidae, Pieridae, Riodinidae, Lycaenidae, Nymphalidae, and Papilionidae) included (a) range of host plant families, (b) main host plant family used, (c) known host plant species, and (d) the most important host family according to Bink (1992). Using this dataset, the complete set of plant families, identified as hosts, was compiled using The Plant List (http://www.theplantlist.org/). In total, 48 plant families are used as hosts by the 145 butterfly species.

### 2.2 Chemical defense characterization

Using the selection of 48 host plant families, a literature search was done to determine their specific secondary metabolite composition to the most detailed level possible. Several search engines were used to search for literature on secondary metabolites consisting of Wageningen University Global Search, Elsevier Scopus, and Web of Science. Search queries were constructed as follows: “secondary AND metabolites AND Asteraceae.” Searches were carried out during November and December 2017. Special care was taken to limit the search to vegetative plant tissue, where possible the genus was added to the search query to ensure the most accurate representation of metabolite profile for each butterfly–host interaction. This is especially important in cases where only a single species or genus functions as host plant, such as in *Humulus lupulus* L. of the Cannabaceae or *Euonymus europaeus* L. of the Celastraceae. To give the most complete chemical defense representation per plant family, searches were carried out in an exhaustive manner, continuing until all literature records of unique secondary metabolite classes were categorized. The secondary metabolites characteristics of all 48 host plant families were compiled using this approach. Information on chemical data was collected for the three main groups of secondary metabolites as classified in Schoonhoven et al. (2005): phenolic, terpenoid, and nitrogen‐containing compounds. Acetylenic compounds were omitted due to their underrepresentation in the available literature. Within this broad classification scheme, the chemical characteristics were identified to metabolite group (e.g., phenolic), type (e.g., flavonoid), class (e.g., flavonol), subclass (flavonol glycoside), and where possible to specific compound (e.g., rutinoside). In further statistical analyses, we included data on higher chemotaxonomic levels, not including records of specific compounds. Chemical defense records (Table S1) were later summarized per plant family as a presence–absence (0,1) trait matrix for further analysis (Table S2). We acknowledge that publication bias may be present in any literature‐based approach; however, we have taken several control measures to minimize the impact thereof. We attempt to correct for publication bias by (a) focussing on plant family level, so aggregating the amount of available literature, (b) using constant search terms, (c) restricting our analysis to vegetative parts only, (d) restricting our analysis to well‐researched main secondary metabolite groups, (e) only using higher chemotaxonomic levels in our statistical analyses, and (f) exhaustively compiling metabolite class records, so fully capturing data from literature records.

### 2.3 Phylogenetic relationships of butterflies and host plant families

To study the interactions of butterflies and host plant families in a network, data on phylogenetic relationships of both have been gathered. The phylogenetic relationships of host plant families were generated by pruning selection of host families (*n* = 46) from a large dated molecular phylogeny presented in Ramírez‐Barahona et al. (2020). For the butterflies, the phylogenetic relationships as inferred by Wiemers et al. (2020) were used and pruned to the selection of studied species. Butterfly species and subfamily naming and taxonomy are reported according to Wiemers et al. (2018). Branch lengths for both the host plant and butterfly phylogeny were given as ages in millions of years (myr).

### 2.4 Chemical defense relationships of host plants

**FIGURE 1:**
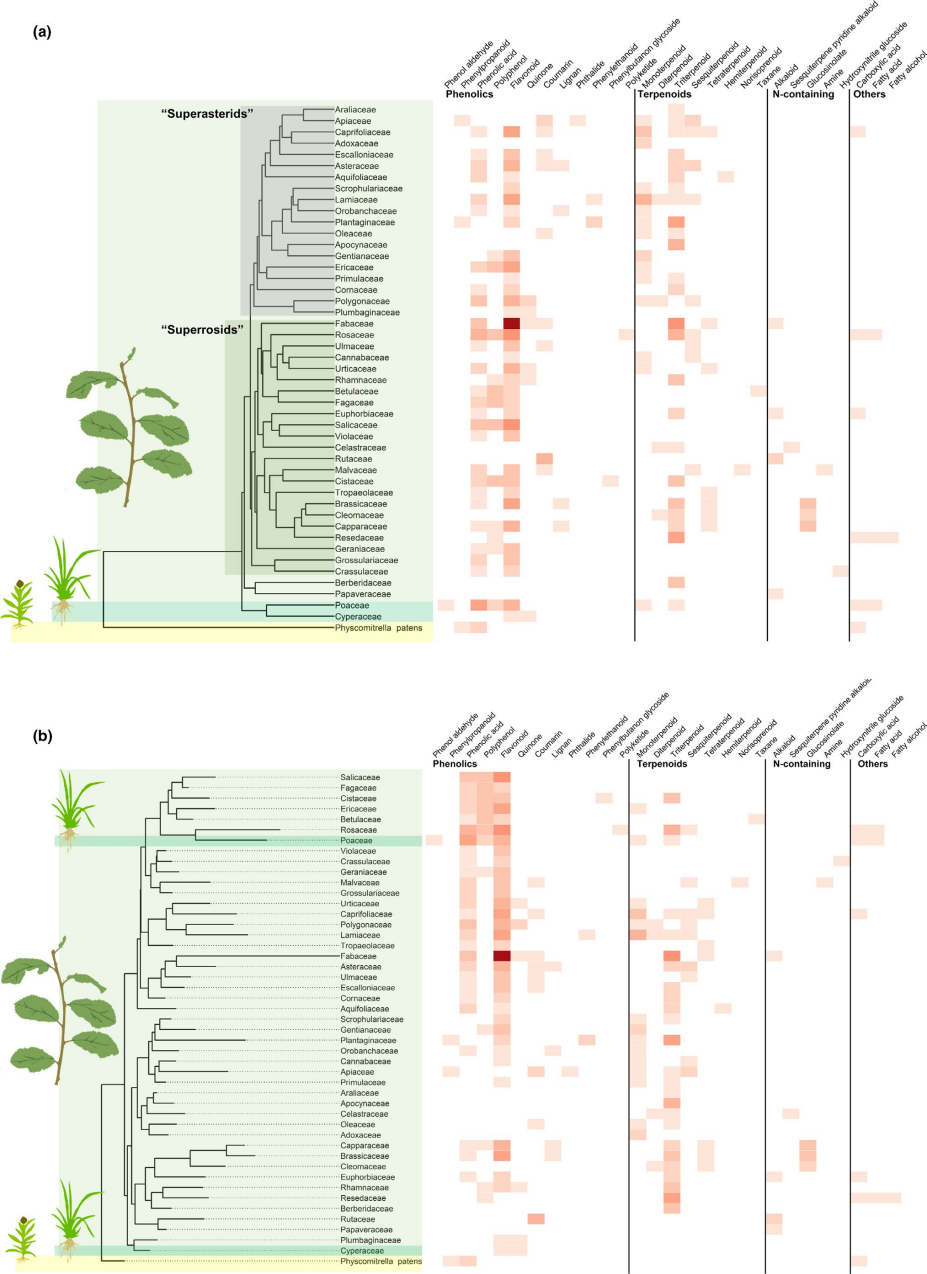
Heatmap of secondary metabolite diversity per butterfly host plant family. Intensity of shading indicates diversity of compounds found in the literature per host plant family, expressed as the number of subclasses per unique secondary metabolite class. Plant icons indicate the following: leaves for eudicots, grass for monocotyledons, and a moss for mosses (a) Host plant families are positioned according to phylogeny following Ramírez‐Barahona et al. (2020). (b) Host plant families are positioned according to chemical defense relationships in a chemical defensogram based on the secondary metabolite matrix in Table S2

To infer chemical defense patterns among host plant families, dendrograms were constructed based on chemical characters, from now on referred to as a “chemical defensograms” after Endara et al. (2017). These chemical defensograms (Figure 1b) were constructed by clustering chemical defense profiles of host plant families (Table S2). Cluster algorithm choice was made by selecting the algorithm that gave the highest correlation between initial Manhattan dissimilarity matrix of the host plants (based on the metabolite matrix Table S2) and a matrix of cophenetic distances between host plants after clustering; correlations are given in Table 1. This approach was used to ensure the chosen cluster algorithm maximizes the retention of the variation in the original dataset and most accurately depicts the relationships among the different taxa (Borcard et al., 2018; Endara et al., 2017). Neighbor‐joining clustering using ape::bioNJ showed the highest correlation between the two dissimilarity matrices (Mantel's r = 0.92) (Paradis & Schliep, 2019). This clustering algorithm was subsequently used for all constructed chemical defensograms. Both the chemical defensogram and the host plant phylogeny were plotted with a heatmap of secondary metabolite diversity to show chemical defense patterns of host plants using ggtree::gheatmap (Yu et al., 2017). Here, the total diversity of metabolites, consisting of 105 presence–absence traits, was condensed into 28 traits which represent secondary metabolite classes (within the main groups: phenolic, terpenoids, nitrogen‐containing, and “others”). The values assigned per trait correspond to the diversity (i.e., number of subclasses) of metabolites found per host plant family in a certain metabolite class. This matrix of secondary metabolite counts (Table S3) was used to illustrate the diversity of metabolites per category, for a certain plant family, with color intensity signifying increasing metabolite counts per unique class (Figure 1b).

**TABLE 1:** Matrix correlations between the original metabolite dissimilarity matrix of host plants and the cophenetic distance matrix of the chemical defensogram, sorted ascendingly by clustering algorithm

Clustering algorithm	Pearson's *R* ^2^
Ward.D2	0.45
Ward.D	0.52
Complete linkage	0.62
WPGMC	0.77
Single linkage	0.82
WPGMA	0.83
UPGMC	0.84
UPGMA	0.85
Neighbor‐joining	0.92

Note

Clustering using the neighbor‐joining algorithm resulted in the highest correlation between the two matrices.

The model moss species *Physcomitrella patens* (Hedw.) Bruch & Schimp. was added as the outgroup to the selection of angiosperm taxa; secondary metabolite information was obtained from the following studies: Erxleben et al. (2012); Ponce de León and Montesano (2017). Although functioning as a food plant for some specialized insect species (Cooper‐Driver, 1978), bryophytes are largely under‐utilized by insect herbivores and particularly Lepidoptera (Gerson, 1969; Glime, 2006; Weintraub et al., 1995). Therefore, we take the model moss *P. patens* to represent a basal form of antiherbivore defense, particularly since Papilionoidea only rarely feed on mosses and their chemical defenses (Singer & Mallet, 1986).

### 2.5 Butterfly assemblage correlation tests

To detect trends in the dissimilarity of the butterfly assemblage and the phylogenetic and secondary metabolite dissimilarity of host plant families, matrix correlation tests were performed using the methodology described by Endara et al. (2017). In detail, Mantel tests were performed for the total butterfly assemblage and in addition for each of the larger butterfly families. Only those families with a substantial number of species and host plant interactions were selected as subsets for the analysis, resulting to following four larger butterfly families: Lycaenidae, Pieridae, Nymphalidae, and Hesperiidae. For all five subsets (total butterfly assemblage and the four larger butterfly families), three separate dissimilarity matrices were calculated. For phylogenetic dissimilarity, we constructed a matrix consisting of the cophenetic distance between families in the host plant family phylogeny. The cophenetic distance was calculated using the function ape::cophenetic.phylo (Paradis & Schliep, 2019). Another dissimilarity matrix was calculated based on the host interactions, between butterfly species and their host plant families; herbivores of a particular plant family are hereafter referred to as “butterfly assemblages.” Here, the Manhattan dissimilarity index was applied to a binary matrix (0,1) describing the interaction of butterflies with the selection host plant families. Finally, a Manhattan dissimilarity matrix was constructed based on the presence–absence (0,1) matrix of secondary metabolites (chemical defense dissimilarity). The Manhattan dissimilarity index was chosen to provide the measure of dissimilarity for the last two matrices as it was most suitable for the data according to vegan::rankindex (Oksanen et al., 2018).

For the total butterfly assemblage and each major butterfly family, two Mantel tests (phylogenetic and chemical defense dissimilarity) were carried out using vegan::mantel (Oksanen et al., 2018). These two tests each return a value for Mantel's r which provides a degree of correlation between the two input matrices, that is, the butterfly assemblage with either (a) dissimilarity of host plant phylogeny or (b) the dissimilarity of host plant chemical defenses. Comparing the resulting test statistic allows for the comparison of the strength of correlation of butterfly assemblages with phylogenetic and with chemical defense relationships of host plant families. An additional analysis was performed using only dicot‐feeding butterflies and their host plant families to separate the possibly different effects on butterfly assemblages by monocotyledonous and dicotyledonous host plant families.

### 2.6 Cophylogenetic analyses

We further examined the possible modes of coevolutionary interactions between butterflies and their hosts. In order to distinguish between phylogenetic coevolution and “chemical defense coevolution,” two cophylogenetic analyses were carried out per interaction network. These analyses report the degree of congruence between phylogenies in an interaction network (Balbuena et al., 2013, 2020; Hutchinson et al., 2017). Phylogenetic congruence of interactions between two interacting clades, that is, interactions between species in similar phylogenetic positions, is considered to indicate shared evolutionary history (Hafner et al., 2003; Herrera et al., 2016; Hutchinson et al., 2017). The complete network consists of 137 butterfly species and 46 host plants, with 236 herbivory interactions. Since the dataset is a complex network, often with multiple links between the two sets of taxa, cophylogenetic tests were carried out using the Procrustean Approach to Cophylogeny (PACo) as proposed by Balbuena et al. (2013) and later adapted for use in R by Hutchinson et al. (2017). PACo analysis was run with a conservative null model; permutations were carried out with vegan's “quasiswap” where the number of interactions per species, and consequently those in the total network, was conserved (Hutchinson et al., 2017). Furthermore, PACo was run with the “symmetric” function, where neither the host nor the herbivore is identified a priori as the driver of evolution of the other. These parameters were chosen to ensure a conservative test (Hutchinson et al., 2017).

A direct comparison of the strength of cophylogenetic signal in phylogenetic relationships versus that in chemical relationships is unreliable due to different branch lengths in the different host plant dendrograms. However, the similarities or differences in patterns encountered between the two can be accurately identified when plotting the cophylogenetic signal of clades in a tanglegram. Here, Random TaPas is a suitable method whereby the cophylogenetic signal between two phylogenies is identified by running a global‐fit model on *n* number subsamples that represent the most cophylogenetic links in the dataset (Balbuena et al., 2020). We used PACo as the global‐fit method and *n*, the number of subset tanglegrams extracted, was set at the maximum *n* allowing for ~100,000 permutations that ensured a sufficient fraction of total interaction links (10%–20%). This was determined using the One2one.f function of Random TaPas (Balbuena et al., 2020). Random TaPas reports a network Gini coefficient (G*) as global test statistic (Balbuena et al., 2020). In a network with multiple interactions per host and symbiont species, a G* of 2/3 or 0.66 is taken as the null hypothesis, a G* from a network with interactions between species display coevolutionary patterns by chance (Balbuena et al., 2020). With an increasing of cospeciation, the network G* approaches 0, and correspondingly as cospeciation decreases, the network G* approaches 1. The weighted frequency residuals of Random TaPas were plotted on the interaction network to visualize the relative degree of congruence per link, using a diverging color scale. All statistical analyses were performed in the R Statistical Environment v.3.6.2 (R Core Team, 2019).

## 3 RESULTS

### 3.1 Host plant chemical defense profiles

Based on our literature search, the resulting metabolite composition table encompasses 647 data points based on 144 literature sources (see Table S1). The table provided data on three levels and in some cases four levels of chemical classification per compound, preserving the chemotaxonomic relationships and consequently the complexity of metabolite composition. For two host plant families (Juncaceae and Lythraceae), no secondary metabolite information could be found, and these were omitted from further analysis resulting in a total of 46 host plant families. Both omitted families are minor host plant families and only two butterfly species in the analysis use them as host plants (Table S4).

The chemical defensogram (Figure 1b) and the host plant phylogeny (Figure 1a) heatmaps show that plant families have distinct chemical defense trait signatures and often contain multiple classes of secondary metabolites. Clustering host plants based on phylogenetic relationships and chemical defense traits resulted in widely different tree topologies (Figure 1a,b). Two general clusters of plant families can be loosely identified from the defensogram tree structure, with corresponding patterns of chemical defense traits indicated by the heatmap (Figure 1b). Both clusters are largely defended by phenolic and terpenoid compounds, with N‐containing defenses showing a scattered distribution across the studied plant taxa. Families in the lower cluster generally contain terpenoids belonging to the mono‐ and triterpenoids classes, while families in the upper cluster consistently contain the phenolic classes of phenolic acids and flavonoids. The chemical defensogram illustrates that chemical defense traits do not necessarily seem to be phylogenetically conserved at plant family level and may vary widely among closely related plant families (Figure 1b).

In contrast to the often disparate defense compositions of closely related plant families, some exceptions exist, where the presence of characteristic defenses persists in closely related families (Figure 1b). Notably in the Brassicales, where glucosinolate compounds are found in the Brassicaceae and in close relatives Cleomaceae and Capparaceae. Fabaceae was found to have the largest diversity of flavonoid compounds.

Based on Figure 1, we find distinct chemical defense signatures per host plant family. Comparison between the phylogenetic heatmap (Figure 1a) and the chemical defensogram heatmap (Figure 1b) shows that these do not reflect phylogenetic relatedness and therefore do not necessarily indicate that closely related plant families have greatest overlap in their chemical defense suite.

### 3.2 Butterfly assemblage matrix correlation

**FIGURE 2:**
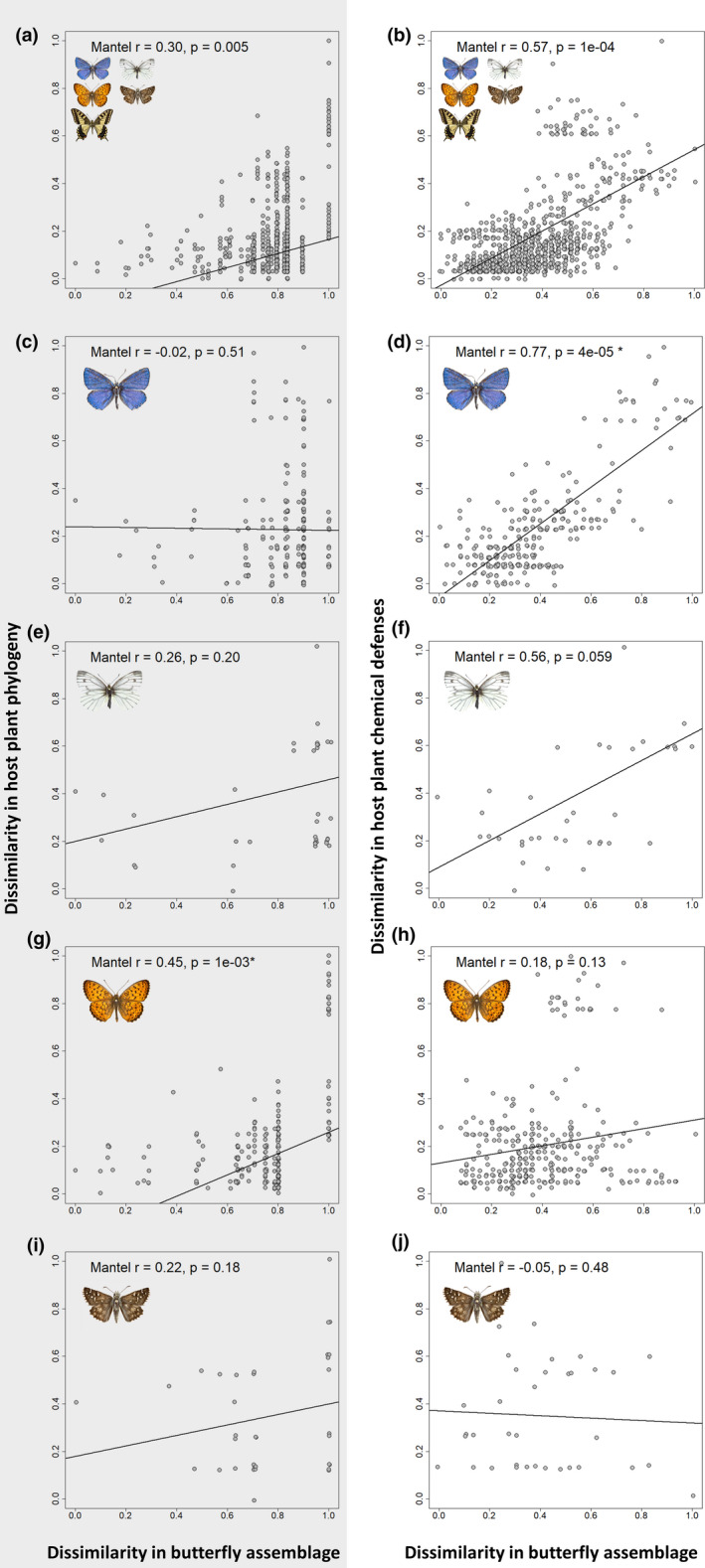
Matrix correlations of the total interacting community (a, b), Lycaenidae (c, d), Pieridae (e, f), Nymphalidae (g, h), and the Hesperiidae families (i, j). Left column (a, c, e, g & i) shows the dissimilarity in butterfly species assemblage plotted against the dissimilarity in phylogeny for each pairwise combination of host plant family. Right column (b, d, f, h & j) shows the dissimilarity of butterfly species assemblage plotted against the dissimilarity in chemical defenses for the same selection of host plants

**FIGURE 3:**
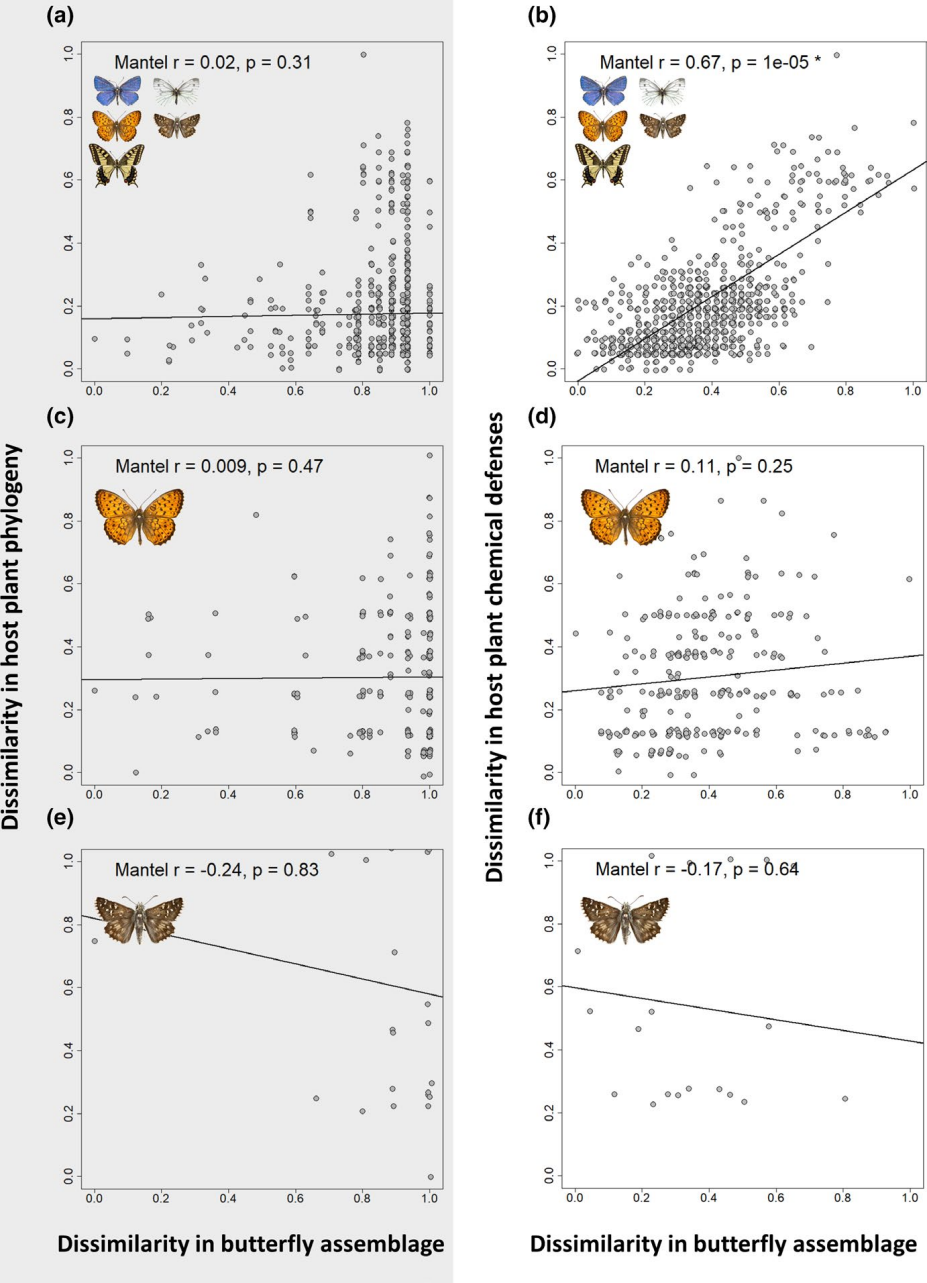
Mantel test results after removing monocot families (Poaceae & Juncaceae) and monocot feeders. Matrix correlations of the total interacting community (a, b), Nymphalidae (c, d), and the Hesperiidae families (e, f). Left column (a, c & e) shows the dissimilarity in butterfly species assemblage plotted against the dissimilarity in phylogeny for each pairwise combination of host plant family. Right column (b, d & f) shows the dissimilarity of butterfly species assemblage plotted against the dissimilarity in chemical defenses for the same selection of host plants

Matrix correlations between butterfly herbivore communities and the host plant families revealed the importance of chemical defenses in determining herbivore assemblages (Figure 2 & Table 2). The most significant pattern was observed from analysis of the total butterfly–host community of northwestern Europe, consisting of 145 species interacting with 46 host plant families (Figure 2a,b). We found strong positive correlation between butterfly assemblages and host plant chemical defenses (Mantel *r* = 0.57, *p* < 0.001) (Figure 2b) while weaker correlation was found between butterfly assemblages and plant phylogeny (Mantel *r* = 0.30, *p* = 0.005) (Figure 2a). Upon removal of monocot families (Poaceae & Cyperaceae) and their butterfly assemblage from the analysis, the result became even more pronounced (Figure 3a,b). Removal of these taxa resulted in stronger positive correlation between butterfly assemblage dissimilarity and host plant chemical dissimilarity (Mantel *r* = 0.67, *p* < 0.001) (Figure 3b). However, we found no correlation between butterfly assemblage dissimilarity and host plant phylogenetic dissimilarity when omitting monocot families (Mantel *r* = 0.02, *p* = 0.31) (Figure 3a).

**TABLE 2:** Matrix correlations between lepidopteran herbivores and their host plant families

Butterfly family	Phylogenetic correlation		Chemical defense correlation		Number of butterfly species	Number of host plant families
	Mantel r	*p*‐value	Mantel r	*p*‐value		
Total	0.30	5e−03*	0.57	1e−04*	145	46
Lycaenidae	−0.02	0.51	0.77	4e−05*	43	24
Pieridae	0.26	0.20	0.56	0.059	13	9
Nymphalidae	0.45	1e−03*	0.18	0.13	66	26
Hesperiidae	0.22	0.18	−0.05	0.48	18	9

Note

Given are Mantel correlation coefficient (Mantel *r*), the corresponding *p* value, and the size of the host and herbivore communities that are compared in the analysis. Statistics for both correlation with phylogenetic and chemical defense similarity of hosts are presented.

* Significant (*p* < 0.05) *p* values are indicated

We also performed detailed analysis for specific butterfly family host plant networks. Of the four butterfly families with a substantial number of species and host plant interactions (Lycaenidae, Pieridae, Nymphalidae, and Hesperiidae), two families showed strong positive correlation with host plant chemical defense (Table 2, Figure 2d,f). The Lycaenidae network consisted of 43 butterfly species interacting with 24 host plant families. Again, a strong positive correlation was seen between the dissimilarity in host plant chemical defense composition and that of the lycaenid butterflies (Mantel *r* = 0.77, *p* < 0.001) (Figure 2d). No correlation was found between the phylogenetic dissimilarity of the host plants and that of the corresponding Lycaenidae butterfly assemblages (Mantel *r* = −0.02, *p* = 0.51) (Figure 2c).

We identified similar patterns as described above in the Pieridae network (Table 2, Figure 2e,f). This network consisted of 13 butterfly species on 9 host plant families in the analysis. Although the Mantel tests did not return statistically significant results (*p* < 0.05), *p*‐values close to the significance margin were reported. Strong positive correlation was found between chemical defense dissimilarity and pierid butterfly assemblage dissimilarity (Mantel *r* of 0.56, *p* = 0.059) (Figure 2f). Weak, positive correlation between host plant phylogenetic dissimilarity and dissimilarity in Pieridae butterfly assemblage was statistically insignificant (Mantel *r* of 0.26, *p* = 0.20) (Figure 2e).

**FIGURE 4:**
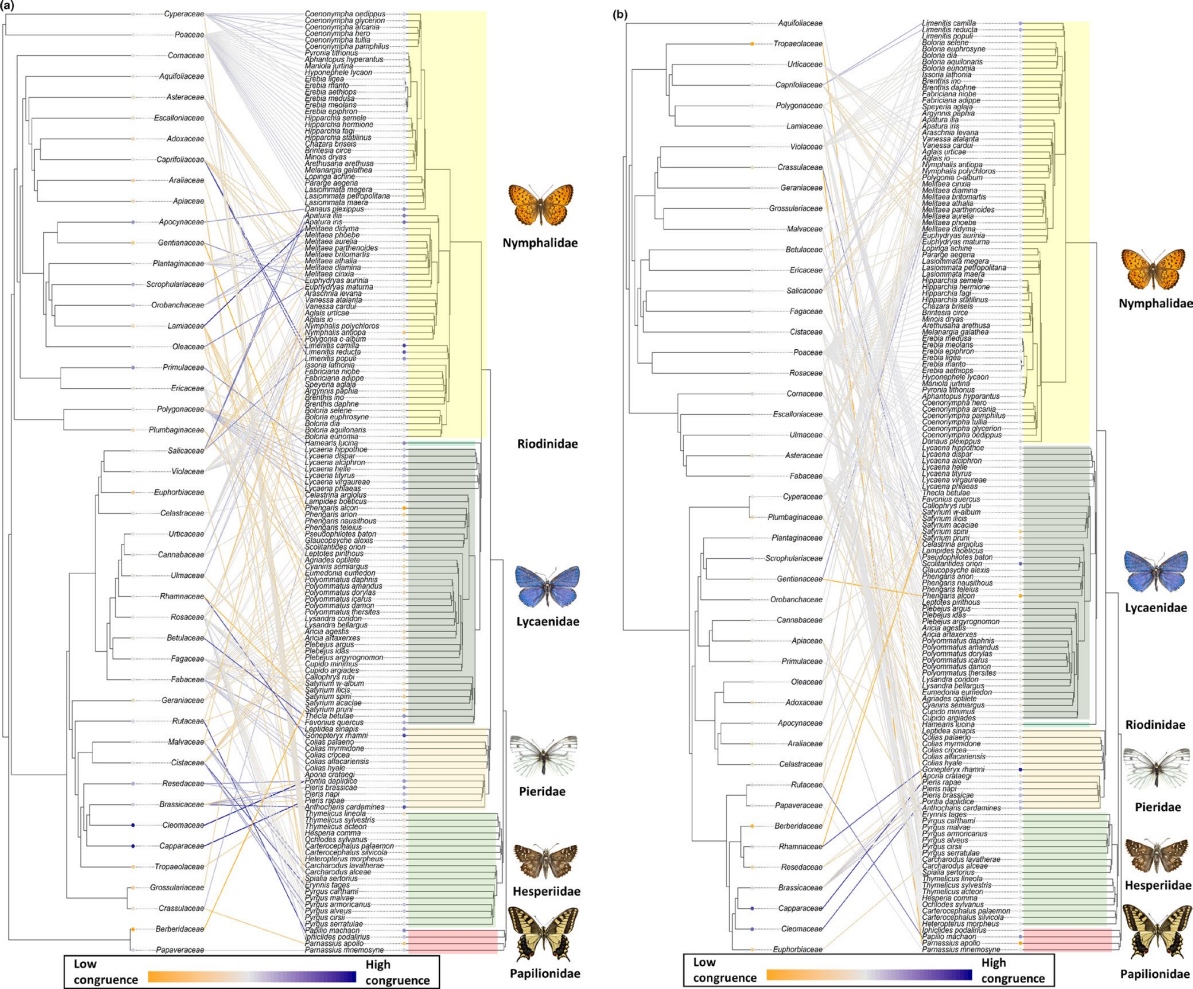
The total butterfly–host plant community, showing herbivory interaction links with their host plant families. Butterfly species are positioned according to Wiemers et al. (2020). The degree of congruence between the two trees reported by Random TaPas is plotted as a heat signal, indicating links with minimum congruence (yellow) through neutral (gray) to maximally congruent links (blue). (a) Host plant families are positioned according to phylogeny following Ramírez‐Barahona et al. (2020). (b) Host plant families are positioned according to chemical defense relationships in a chemical defensogram

A strong positive correlation was recorded between the Nymphalidae assemblage and host plant phylogenetic dissimilarity (Table 2, Figure 2g). However, no significantly positive correlation was found between nymphalid butterfly assemblage and chemical defense dissimilarity of their host plant families (Table 2, Figure 2h). Nymphalidae constitute a large butterfly family in the dataset (*n* = 66 spp.); however, the majority (*n* = 30 spp.) is made up of the monocot feeding Satyrinae subfamily. Within the Nymphalidae, the Satyrinae species use monocotyledonous hosts in the families Poaceae, Cyperaceae, and Juncaceae (Tables S4 & S5, Figure 4a,b). Species from the other subfamilies show varying degrees of host family specificity (Figure 4a,b). Particularly, the Heliconinae and the Nymphalinae, which make up the bulk of the species (30 spp.), feed on a large number of host plant families. When removing monocot feeding species, correlations between assemblage dissimilarity and host plant phylogenetic dissimilarity as well as host plant chemical defense dissimilarity disappeared (Figure 3c,d).

Hesperiidae was the only clade within total butterfly assemblage that neither showed correlation of butterfly assemblage composition with host plant phylogenetic dissimilarity (Table 2, Figure 2i) nor host plant chemical defense dissimilarity (Table 2, Figure 2j). The Hesperiidae also showed a relatively low diversity of host plant interactions. Nearly half of the species, five Hesperiinae species and three Heteropterinae species, feed on three closely related monocotyledonous host plant families; the Juncaceae, Cyperaceae, and the Poaceae (Tables S4 & S5, Figure 4a,b). The other 10 species use host plants belonging to the Rosaceae, Malvaceae, Cistaceae, Fabaceae, and Lamiaceae (Tables S4 & S5, Figure 4a,b). After removing monocot feeding species, Mantel tests again only reported insignificant results (Figure 3e,f). In summary, few host plant family interactions were present and the majority of the butterflies (13/18 spp.) feed from only one of two distantly related families: Poaceae or Rosaceae.

### 3.3 Cophylogenetic patterns

#### 3.3.1 Total interaction community

**FIGURE 5:**
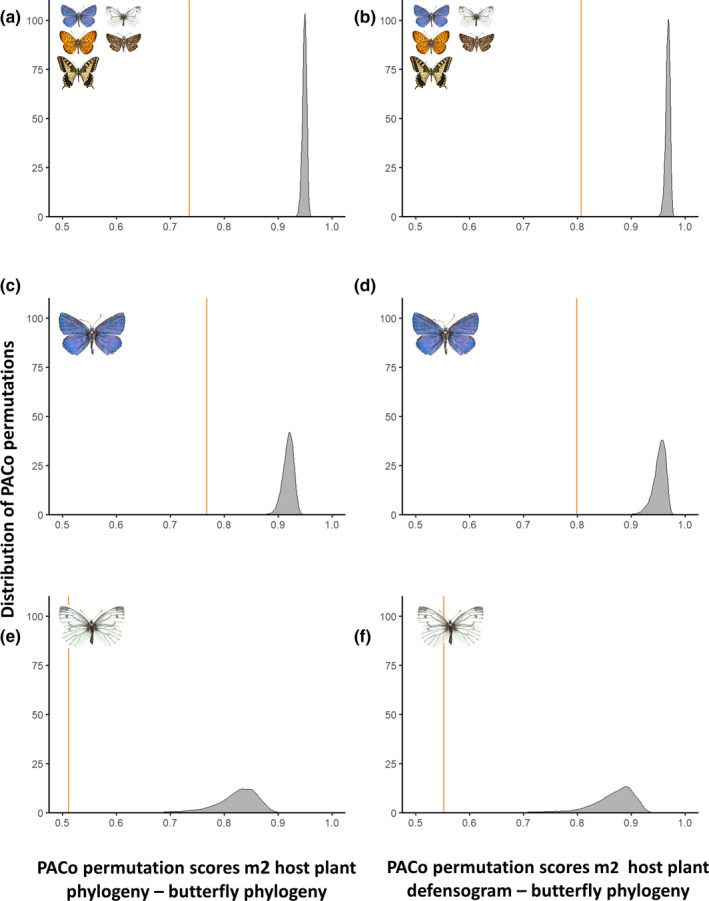
PACo permutation scores of the total interacting community (a, b), Lycaenidae (c, d), and the Pieridae families (e, f). Left column (a, c & e) shows the test output of butterfly phylogeny interacting with host plant family phylogeny for each interaction network. The right column (b, d & f) shows the test output of butterfly phylogeny interacting with host plant family chemical defensogram for each interaction network. The orange line shows the observed best fit Procrustean superimposition (*m* ^2^ *_xy_*) between the actual butterfly & host trees and observed interaction links. The gray distribution shows the distribution of permutation best fit Procrustean superimpositions. For each of the six tests, PACo tests returned statistically significant results (*p* < 0.001). Meaning that for each of the networks tested, the network as observed in nature is statistically more congruent than 10,000 networks based on the same tree topologies but with randomized interaction links

The complete interaction network contained 236 unique butterfly–host plant interactions. The PACo global‐fit analyses indicated significant congruence between butterfly phylogeny and with both, host plant phylogeny (PACo *m* ^2^ *_xy_* = 0.74, *p* < 0.001) and chemical defense composition (PACo *m* ^2^ *_xy_* = 0.81, *p* < 0.001) (Figure 5a,b). Visual assessment of cophylogenetic links between butterfly and host plant taxa showed a lack of clear patterns in the dataset, both when testing between host plant phylogeny and host plant chemical defenses (Figure 4a,b). Global G* coefficients reported by Random TaPas showed low global congruence between host plant phylogeny and butterfly phylogeny, with a normalized Gini coefficient (G*) of 0.77 (Figure 4a). Testing for global congruence between for host plant defensogram and butterfly phylogeny reported G* of 0.82 (Figure 4b). For both networks, few highly congruent links were scattered between a majority of links with low congruence, as indicated by the relatively high global G* values.

#### 3.3.2 Lycaenidae: blues, coppers, and hairstreaks

**FIGURE 6:**
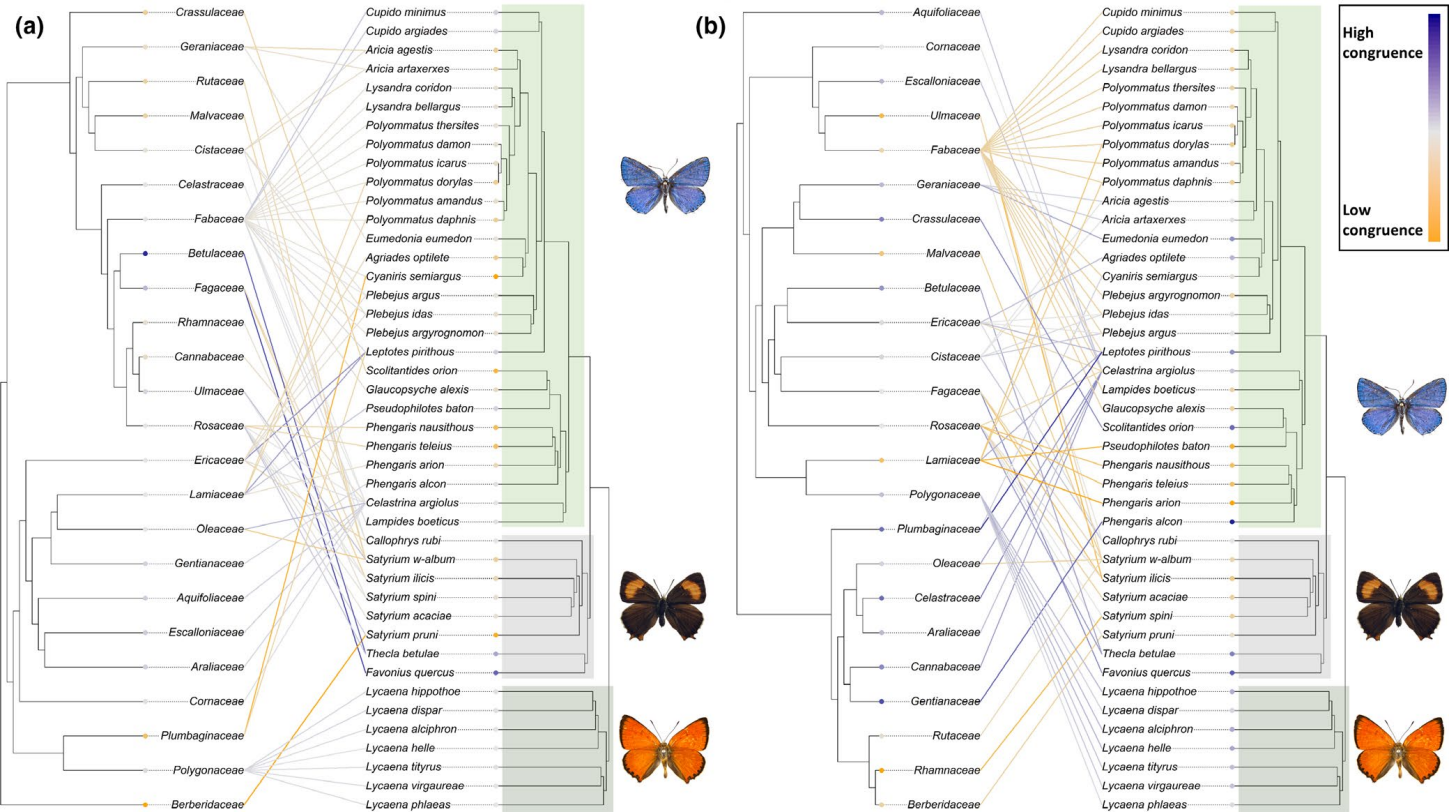
The Lycaenidae–host plant community, showing herbivory interaction links with their host plant families. Lycaenidae species are positioned according to Wiemers et al. (2020). The degree of congruence between the two trees reported by Random TaPas is plotted as a heat signal. The Random TaPas frequency residuals are plotted for each link indicating minimum congruence (yellow) through neutral (gray) to maximally congruent links (blue); color scale is relative to the network. (a) Host plant families are positioned according to phylogeny following Ramírez‐Barahona et al. (2020). (b) Host plant families are positioned according to chemical defense relationships in a chemical defensogram

The lycaenid–host plant interaction network was made up of 75 herbivore–host plant links. For the Lycaenidae family, PACo global‐fit analyses between butterfly phylogeny and host phylogeny indicated statistically significant congruence (PACo *m* ^2^ *_xy_* = 0.77, *p* < 0.001) (Figure 6c). Similar results returned when testing the Lycaenid phylogeny with the host plant defensogram (PACo *m* ^2^ *_xy_* = 0.80, *p* < 0.001) (Figure 5d). Random TaPas analysis returned global G* = 0.73 for the lycaenid and host plant phylogeny network, indicating low global congruence in the network (Figure 6a). Random TaPas particularly reported links of low congruence between the Lycaeninae and Polyommatinae species and their host plants.

Examination of the interaction network of the host plant chemical defensogram with lycaenid butterfly phylogeny yielded several highly congruent links, scattered between links of low congruence (global G* = 0.68) (Figure 6b). All Lycaeninae (coppers) species showed low‐to‐moderate congruence with their host plants based on the chemical defensogram (Figure 6b). This subfamily consists solely of members of the genus *Lycaena* in this study, all feed exclusively on the Polygonaceae, containing mainly phenolic acids, flavonoids, and mono‐, di‐, and sesquiterpenoids (Figure 1b). Within Theclinae (hairstreaks), only two species, *Thecla betulae* L. and *F* *avonia quercus* L., show congruent interaction links with chemical defenses of their host plant families (Figure 6b). *T. betulae* predominantly feeds on woody species in the Rosaceae, containing phenolic acids, polyphenols, and flavonoids (Figure 1b). The same dominant classes of chemical defenses are found in Fagaceae, the host plant family of *F. quercus*.

Congruent links were reported between several Polyommatinae species and host chemical defenses (Figure 6b); however, there was considerable variation. Where Random TaPas reported congruent butterfly–host plant links, for example, links with Gentianaceae, Crassulaceae, Geraniaceae, and Ericaceae, the chemical defense profiles are dominated by phenolic acids and polyphenols, which are tannin‐like compounds and tannic acids.

#### 3.3.3 Pieridae: whites and sulphurs

**FIGURE 7:**
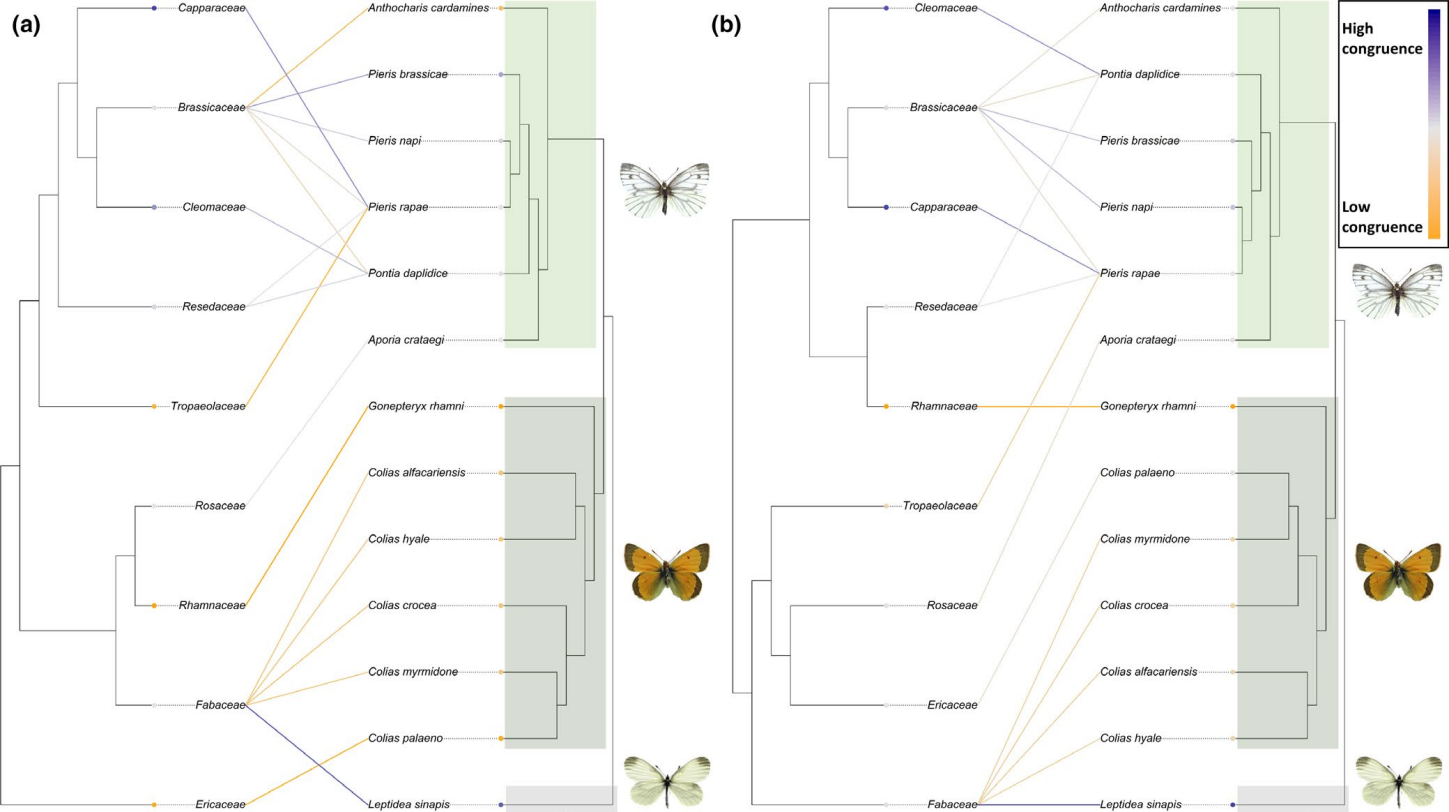
The Pieridae–host plant community, showing herbivory interaction links with their host plant families. Pieridae species are positioned according to (Wiemers et al., 2020). The degree of congruence between the two trees reported by Random TaPas is plotted as a heat signal, indicating links with minimum congruence (yellow) through neutral (gray) to maximally congruent links (blue). (a) Host plant families are positioned according to phylogeny following Ramírez‐Barahona et al. (2020). (b) Host plant families are positioned according to chemical defense relationships in a chemical defensogram

The interaction network of Pieridae and their host plants was made up of 18 herbivore–host plant links. Similar to the total network, statistically significant congruence was again detected by PACo in both networks, Pieridae phylogeny–host phylogeny (PACo *m* ^2^ *_xy_* = 0.51, *p* < 0.001), and Pieridae phylogeny and host defense composition (PACo *m* ^2^ *_xy_* = 0.55, *p* < 0.001) (Figure 5e,f). Random TaPas analysis returned the global G* = 0.67 for the pierid and host plant phylogeny network (Figure 7a). Main pierid host plant families were the Fabaceae for the Coliadinae subfamily and Brassicaceae for the subfamily Pierinae. Congruent links were detected in the pierid–host phylogeny interaction network (Figure 7a). Predominantly, links between host plant families within the Brassicales and pierid species of the subfamily Pierinae were identified as congruent by the Random TaPas analysis. In addition, the Dismorphinae species *Leptidea sinapis* L. and Fabaceae were found to share a congruent interaction link.

The interaction network of Pieridae and its set of host plants clustered on metabolite composition showed more congruent links, Random TaPas reported low global congruence G* = 0.70 (Figure 7b). Congruence was again found for the link between *L. sinapis* and Fabaceae. Weakly congruent links were also identified between Brassicaceae and *Pieris brassicae* L. and *Pieris napi* L. and between *Pieris rapae* L. and the Capparaceae. Another congruent link was detected between *Pontia daplidice* L. and the Cleomaceae. All other links between pierid species and host plant defenses showed low congruence.

## 4 DISCUSSION

While secondary metabolite classes generally show a discontinuous distribution across plant clades (Becerra, 1997; Farrell et al., 1991; Wink, 2003, 2008), they are generally conserved at plant family level (Schoonhoven et al., 2005; Volf et al., 2015; Wink, 2008), often lacking a phylogenetic signal across deeper plant phylogeny (Becerra, 1997; Farrell et al., 1991; Wink, 2003, 2008). Overlap in chemical defense traits in distantly related families may be due to convergence, when similar defense traits have evolved independently a number of times (Whitfeld et al., 2012). These patterns are clearly visible in Figure 1, where plant families have considerably different positions in the phylogeny (Figure 1a) and the defensogram (Figure 1b). Within the eudicot clade, two dominant groups of chemical defenses can be loosely identified from the heatmap patterns (Figure 1a). The superrosids in the analysis comprise families that contain chemical defense compounds across all three large secondary metabolite classes. On the contrary, superasterids seem to be largely defended by classes of phenolics and terpenoid compounds. These two clades, with different compositions of defense traits, allude to the presence of defense syndromes at higher taxonomic levels (Agrawal & Fishbein, 2006).

Northwestern European butterflies show diverse patterns of host plant use, with many butterfly species making use of multiple distantly related plant families (Figure 4a). In this study, we investigated if these host plant patterns are more closely correlated with the presence of shared chemical defenses of hosts than host plant phylogeny. Host plant families with similar metabolite composition showed a significantly greater overlap in butterfly herbivore assemblage (Table 2, Figure 2b); this applies particularly to the eudicots (Figure 3b). Although global patterns were not mirrored in each butterfly family examined (e.g., Table 2, Figure 2h, Figure 3d). These results indicate that similarity in chemical defense composition of host plant families seems to be a more important driver for overall herbivore assemblage similarity than shared evolutionary history of host plants, that is, phylogenetic relationships. Additionally, using different statistical tests, we detected signals of congruence between butterflies and host plant families (Figure 4a,b, Figure 5). These signals of congruence were also detected with chemical defense relationships at host plant family level (Figure 4b, Figure 5b). Again, detailed examination of both phylogenetic and defense networks on a finer level yielded a spectrum of congruence, with both interactions of low and higher congruence within a certain network (Figure 6, Figure 7).

### 4.1 An overview of the total interaction community

Strong positive correlation of butterfly assemblages to plant secondary metabolite composition has been found in a number of host plant genera–moth family interaction networks (Endara et al., 2017) and in other insect herbivore communities (Becerra, 1997; Endara et al., 2018). Especially in specialist insect clades, secondary metabolite similarity was found to be a better predictor for feeding intensity than phylogenetic relatedness of host plants (Becerra, 1997; Pearse & Hipp, 2009; Rapo et al., 2019; Rasmann & Agrawal, 2011). In this study, we confirm this pattern at a macroevolutionary scale by using a large sample of northwestern European butterfly species (145 species) and interactions with host plants at family level. The positive correlations of chemical defensogram and butterfly assemblages (Table 2, Figure 2) confirm the importance of shared chemical defenses for plant–butterfly interaction networks. Furthermore, we show that overlap in chemical defense traits is likely to be more important than phylogenetic relatedness in determining plant–butterfly interaction networks at a macroevolutionary scale.

### 4.2 Implications for insect–plant community ecology

Classical ecological hypotheses state that higher plant diversity predicts higher herbivore diversity (Crutsinger et al., 2006; Haddad et al., 2001; Hutchinson, 1959; Siemann et al., 1998). However, plant phylogenetic alpha diversity within natural communities in European grassland systems was found to be a rather poor predictor of butterfly phylogenetic alpha diversity (Pellissier et al., 2013). This implies that phylogenetic measures of plant diversity do not necessarily reflect the total functional space available to herbivores within a community and that other measures, such as the diversity of chemical defense strategies, are likely to be a better predictor (Pellissier et al., 2013). Our results also show that phylogenetic measures of plant diversity do not necessarily capture the functional space available for butterflies (Table 2, Figure 2, Figure 3a,b).

We find that similarity in chemical defenses of host plant families results in similar butterfly assemblages, while phylogenetic relatedness poorly predicts similarity in butterfly assemblages (Figure 2, Figure 3a,b). Consequently, higher diversity of chemical defense strategies in a community would allow for more diversity in butterfly assemblages. Becerra (2015) finds such strong positive correlations between butterfly assemblage diversity and plant antiherbivore trait diversity. Similar results have been shown on a finer scale for other insect herbivores by Richards et al. (2015) and by Becerra (1997). However, phylogenetic diversity should not be disregarded, since the inclusion of more families increases the probability of including a family with higher chemical dissimilarity and so the potential for higher diversity in the resulting butterfly assemblage.

The existence of a direct positive relationship between increasing diversity and increasing specialization is considered a classical ecological hypothesis (Hutchinson, 1959). According to our results, this relationship may be more evident when considering increasing diversity in chemical traits rather than absolute phylogenetic diversity. Previous studies also supported the hypothesis that elevated diversity in chemical defenses in a plant assemblage allows for greater number of specialized herbivory niches and predicts a higher diversity of herbivorous insects within a community (Becerra, 2007, 2015; Dyer et al., 2007). This is also known as the “Resource Specialisation Hypothesis,” with chemical defenses forming the resources that herbivorous insects specialize on (Keddy, 1984; Moreira et al., 2016). Furthermore, the effect on higher chemical defense diversity may positively influence the evolution of specialization, since host switching becomes increasingly difficult as chemical defenses diverge (Ehrlich & Raven, 1964; Richards et al., 2015).

Our results point to chemical defenses as an important measure of functional space available to insect herbivore diversity within a community. Diverse communities of plants, containing plant families with each a different composition of chemical defenses, support diverse assemblages of insect herbivores. Such communities are also likely to show gradually increasing levels of host plant specialization over time. When assessing patterns of diversity across and within plant–insect interactions, incorporating plant trait diversity, such as the diversity in chemical defense traits, can provide new insights into patterns of insect diversity.

### 4.3 Coevolutionary patterns in butterfly families

Of the four butterfly families examined in detail, two, the Lycaenidae and Pieridae, showed strong positive correlation with chemical defenses (Table 2, Figure 2) and were therefore selected to further identify the chemical defense basis behind this correlation.

#### 4.3.1 Lycaenidae: blues, coppers, and hairstreaks

Overall, Lycaenidae species were found to have congruent interaction links with their host plant chemical defenses (Figure 6b). All feed on plants that are broadly characterized by phenolic defenses: phenolic acids, polyphenols, and flavonoid compounds (Figure 1b). These compounds are generally tannins, or precursors and derivatives thereof. Phenolics, and especially tannins, are commonly present in the chemical defense suite of plants with a woody habit (Barbehenn & Constabel, 2011), but they are not limited to woody growth forms and are known to be antiherbivore defenses in herbaceous species of Fabaceae (Goverde et al., 1999; McMahon et al., 2000). Tannin‐feeding insects have developed numerous physiological and morphological adaptations to be able to feed on tannin‐containing plants (Barbehenn & Constabel, 2011). Adaptation to a tannin enriched diet has been shown for a number of lepidopteran species as well as toxicity symptoms for species that are not adapted to tannin‐rich host plants (Berenbaum, 1983; Karowe, 1989).

The majority of the Polyommatinae are Fabaceae feeding; however, links with the chemical defenses of this host plant family appear to be incongruent (Figure 6b). *Polyommatus icarus* Rottemburg has been shown to sequester flavonoids from Fabaceae, mainly in their wings at different concentrations in males and female adults (Schittko et al., 1999). Another species, *Polyommatus bellargus* Rottemburg, has also been shown to sequester dietary flavonoids in the wings (Geuder et al., 1997). Both authors suggest a role of wing flavonoids in mate recognition of *Polyommatus* (Geuder et al., 1997; Schittko et al., 1999). If flavonoids play a role in mate recognition, then it is likely that flavonoid‐rich host plants are a requirement for *Polyommatus* species and that these species have been interacting with flavonoid‐rich host plants throughout their evolution. However, our results do not show congruent links between *Polyommatus* spp. and fabaceous host plants (Figure 6b).

It has been suggested that patterns of host use by the Lycaenidae are, to some degree, determined by symbiotic relationships with ants (Fiedler, 1994). In general, the subfamily Lycaeninae does not seem to form ant associations and even lacks dorsal nectary organs (Fiedler, 1991). The Theclinae family is facultatively associated with ants; containing species with low levels of myrmecophily and a reduction of ant‐association organs (Fiedler, 1991). In the Polyommatinae, nearly all species have ant associations (Fiedler, 1991). In general, myrmecophilous species have broader host ranges, even at family level, than myrmecoxenes (Fiedler, 1994). However, there are also ample examples of obligate myrmecophiles with highly restricted host plant use, such as the *Phengaris* species in this analysis (Witek et al., 2008). Our results provide little evidence of structuring due to ant interactions, as congruent links are scattered throughout the lycaenid phylogeny.

#### 4.3.2 Pieridae: whites and sulphurs

Examination of the interaction network based on host plant phylogeny, few congruent links between Pierinae (whites) and their host plants could be identified (Figure 7a). Almost all host plants belong to the order Brassicales: Cleomaceae, Brassicaceae, Resedaceae, Capparaceae, and Tropaeolaceae. In comparison, links between three *Pieris* species were found congruent in the interaction network based on host plant chemical defenses (Figure 7b). These are between *P*. *brassicae* and *P. napi* and Brassicaceae and *P*. *rapae* and Capparaceae. *Pieris* butterflies are well known for their specialist lifestyle on brassicaceous plants, and their ability to detoxify highly toxic glucosinolate compounds (Wittstock et al., 2004). Coevolution of Brassicales glucosinolate chemical defenses and Pierinae butterflies has been shown to occur via the arms‐race model of Ehrlich & Raven (Edger et al., 2015). However, our results do not show these clear overall cophylogenetic patterns as expected. Indeed, congruent links were only shown for a limited subset of the *Pieris* species in the analysis (Figure 7a,b).

In our analysis, relatively low levels of congruence between Pierinae and their host plant defenses indicate that additional defense traits may be causing coevolutionary patterns between the Pierinae and Brassicales. Due to the ability of specialist *Pieris* spp. to successfully detoxify the main chemical defense of brassicaceous plants, other forms of defense have evolved (Schoonhoven et al., 2005). Recent studies on this group have shown that Brassicaceae have evolved diverse antiherbivore defense traits against pierid herbivores, including the attraction of butterfly–parasitoids and egg‐killing necrosis reactions (Fatouros et al., 2014; Griese et al., 2019). In addition, Brassicaceous trichomes may be important in antiherbivore defense (Beilstein et al., 2006), such as in *Arabidopsis thaliana* (L.) Heynh., where branched trichomes have been shown to be important as antiherbivore defense against a specialist lepidopteran (Handley et al., 2005). Such structural defenses may also play a role in determining coevolutionary patterns between plants and their herbivores, which has been shown in different herbivore–host plant systems (Cardoso, 2008; Rathcke & Poole, 1975).

Aside from the Pierinae, further congruent links were also detected based on host plant phylogeny between *L*. *sinapis* and Fabaceae (Figure 7a). Fabaceae is found to be the ancestral host plant of the Pieridae (Braby & Trueman, 2006). In the chemical defensogram network, the link between *L. sinapis* and Fabaceae also showed congruence, indicating a relationship with Fabaceae chemical defenses (Figure 7b).

#### 4.3.3 Nymphalidae and Hesperiidae: brush‐footed butterflies and skippers

Globally, the Nymphalidae are known to contain subfamilies with both restricted and broad host plant associations (Ferrer‐Paris et al., 2013). This holds true in our analysis. Janz et al. (2006) proposed the “oscillation hypothesis” for Nymphalidae host specialization, where repeated oscillations and specializations are thought to be the driving force behind this pattern. Examination of an interaction network in the Swiss alps by Pellissier et al. (2013) found no correlation in the diversity of plant species assemblages with the diversity of Nymphalidae species assemblages. This was explained by the presence of a high degree of specialization and, hence, narrow niche breath of a large subfamily (Satyrinae) in combination with the largely polyphagous nature of the other species within the family. Our results indicate a strong, significant correlation of nymphalid assemblage when examined based on phylogenetic relationships, and a weaker correlation when examining host plant chemical defenses (Figure 2g,h). However, this pattern seems to be entirely driven by the monocot specialized Satyrinae clade. Since without monocotyledonous plant families and their associated satyrinids, no correlation could be detected with either host plant phylogenetic or chemical defense dissimilarity (Figure 3c,d). This could be due to the monocotyledonous feeding group being specialized to disarm their host plant primary structural defenses: silicates (Alhousari & Greger, 2018; Massey et al., 2006). And that these structural defenses may form a larger barrier for herbivory than monocot chemical defenses. The shift to monocotyledonous hosts between 23 and 36 myr ago has enabled Satyrinae to radiate and become the most speciose nymphalid subfamily (Peña & Wahlberg, 2008). The Nymphalinae and the Heliconinae tend to feed on a large number of distantly related plants (for example *Melitaea* species in Tables S4 & S5, Figure 4a). Host plant use in the *Melitaea* species has been shown to be closely tied to the presence of dietary iridoids, functioning both as oviposition clues (Nieminen et al., 2003) and as predator defenses (Lampert & Bowers, 2010). Wahlberg (2001) showed that the presence of iridoids more consistently predicts host use than host phylogenetic relatedness for the Melitaeini tribe. In sum, the interactions with iridoid glycosides have been well studied in the Melitaeini and serve as an example that chemical specialization across host plant families has occurred within the European Heliconinae. However, this pattern seems to be obscured when analyzing the Nymphalidae at a macroevolutionary scale. Janz et al. (2001) found that multiple repeated patterns of diversification and specialization occurred in the Nymphalini (*Aglais*, *Vanessa*, *Polygonia*, *Nymphalis,* and *Araschnia* sp.) and concluded that these host ranges are dynamic. The high diversity in patterns, with high degrees of specialization occurring along with polyphagy, within the Nymphalidae may cause the ambiguous patterns observed in our study when examining chemical defense and phylogenetic relationships of host plant families and their butterfly herbivores.

Ferrer‐Paris et al. (2013) reported congruent links between the Hesperiinae and Heteropterinae and their dominantly Poales host plants. No congruent links were found between the Pyrginae and their host plant orders. They suggested that monocots are the ancestral host plant lineage for this family. However, because of diverse host use patterns in the basal Hesperiidae, the ancestral character state is difficult to identify (Ferrer‐Paris et al., 2013). Successful adaptation to feeding on Poales, coupled to the ability to cope with their antiherbivore defenses, have allowed for the radiation of monocot feeding Hesperiidae (particularly Hesperiinae) during the Oligocene (40 myr ago) (Sahoo et al., 2017). Aside from the large portion of Poaceae feeders, the other dominant host family of the Hesperiidae is the Rosaceae (Tables S4 & S5). These host plant families show a large overlap in their chemical defenses (Figure 1b) despite little overlap in the interactions recorded for these families. Therefore, no clear correlation of hesperiid assemblage composition with host chemical defenses could be detected in our analysis. Our results for the Hesperiidae are similar to the results for Nymphalidae and could be due to the same host use specialization patterns. Like Nymphalidae, Hesperiidae contain large highly specialized subgroup occurring alongside a fairly polyphagous subgroup. Adaptation to silicate defenses could also play a role in determining the assemblage composition in this butterfly family. Furthermore, the low amount of Hesperiidae species and overall low host interaction diversity may also obscure host use patterns.

### 4.4 Complexity of antiherbivore defense traits

The focus of our study has largely been on chemical defense; however, there is a whole range of physical defenses that remain unexamined in this framework (Massey & Hartley, 2009; Schoonhoven et al., 2005). Physical defenses have been shown to affect insect herbivory on numerous occasions, see review by Hanley et al. (2007). Additionally, different defense strategies may act in concert on herbivores and should therefore not be treated separately (Agrawal & Fishbein, 2006; Romeo et al., 1996). This should also be the case for combinations of physical and chemical defenses. Plants rarely invest in a single line of defense and multiple traits often occur together as antiherbivore defense in any particular plant (Agrawal, 2007; Agrawal & Fishbein, 2006; Rasmann & Agrawal, 2009). For example, Poaceae are well known to be defended by silicate structures and also contain benzoxazinoid chemical defenses (Massey et al., 2006; Niemeyer, 2009; Wouters et al., 2014). Alhousari and Greger (2018) suggested that they act synergistically, and in combination with plant‐induced volatiles, to reduce feeding damage from herbivorous insects.

In the current study, we attempted to summarize the full suite of chemical defenses per plant family. Hereby, we generalize defense syndromes within each family and omit phylogenetic structuring in traits within host plant families. Our approach, scoring the presence–absence of chemical defenses, simplifies several herbivory interaction aspects. These simplifications were made to examine broad patterns at a macroevolutionary scale; however, we acknowledge that this approach does not fully capture recent advancements in insect–plant interaction studies. For instance, chemical defense concentrations have been shown to influence feeding by herbivorous insects and could contribute to observed patterns in our study (Lankau, 2007). Additionally, there may be interactions between chemical defenses and the environment and phytophagous organisms may also induce of chemical defenses, for both see review by Pavarini et al. (2012). Furthermore, incorporation of herbivore specialism/generalism on the effectivity of chemical defenses (Berenbaum, 1981; Lankau, 2007) or of such specialization on the coevolutionary relationships (Agrawal, 2000) would add to our analysis. Taking into account the full selection of antiherbivore defenses, their specificity, interactions, and the influence of the environment are necessary for a complete view of the complex world of plant–insect coevolution.

## 5 CONCLUSION

Our study emphasizes the importance of chemical plant defenses in the composition of the herbivore assemblage specific to a plant family. Although patterns varied in herbivore family‐level analyses, we show that in the full interaction network increasing dissimilarity of plant chemical defenses corresponds with increased dissimilarity of butterfly species assemblage. Thus, in a certain community the presence of plant families with greater dissimilarity in chemical defense traits allows for greater diversity in butterfly assemblages. Within natural communities, such diversity in plant chemical defenses may be more important for the existence of biodiverse insect assemblages than phylogenetic diversity in plant families.

Coevolutionary patterns between butterflies and their host plants remain difficult to elucidate at higher taxonomic levels. However, our results highlight the importance of chemical defenses in deciphering these patterns, even at a macroevolutionary scale. Future studies should focus on the analysis of whole networks of plants interacting with all herbivores and their complete selection of antiherbivore defenses. Incorporation of such multivariate data is required to encompass the full diversity of interactions and to truly reveal coevolutionary patterns, especially in the case of interaction‐rich communities. Considering the full selection of antiherbivore defenses and their evolutionary relationships will allow for a deeper insight into plant–insect coevolution at a macroevolutionary scale.

## CONFLICT OF INTEREST

The authors declare no conflicts of interest.

## AUTHOR CONTRIBUTIONS

**Corné F. H. van der Linden:** Conceptualization (lead); Data curation (equal); Investigation (lead); Visualization (lead); Writing‐original draft (lead); Writing‐review & editing (lead). **Michiel F. WallisDeVries:** Conceptualization (supporting); Data curation (equal); Writing‐review & editing (supporting). **Sabrina Simon:** Conceptualization (supporting); Investigation (supporting); Supervision (lead); Writing‐original draft (supporting); Writing‐review & editing (supporting).

## Supporting information

Table S1

Table S2

Table S3

Table S4

Table S5

## Data Availability

All data are available as supporting information.
